# Transbilayer phospholipid movement facilitates the translocation of annexin across membranes

**DOI:** 10.1242/jcs.217034

**Published:** 2018-07-19

**Authors:** Sarah E. Stewart, Avraham Ashkenazi, Athena Williamson, David C. Rubinsztein, Kevin Moreau

**Affiliations:** 1 University of Cambridge, Metabolic Research Laboratories, Wellcome Trust-Medical Research Council Institute of Metabolic Science, Cambridge, CB2 0QQ, UK; 2 University of Cambridge, Department of Medical Genetics, Cambridge Institute for Medical Research, Wellcome/MRC Building, Addenbrooke's Hospital, Hills Road, Cambridge CB2 0XY, UK; 3 UK Dementia Research Institute, Cambridge Biomedical Campus, Hills Road, Cambridge, UK

**Keywords:** TMEM16F, Lipid flipping, Protein translocation, Annexin

## Abstract

Annexins are cytosolic phospholipid-binding proteins that can be found on the outer leaflet of the plasma membrane. The extracellular functions of annexin include modulating fibrinolysis activity and cell migration. Despite having well-described extracellular functions, the mechanism of annexin transport from the cytoplasmic inner leaflet to the extracellular outer leaflet of the plasma membrane remains unclear. Here, we show that the transbilayer movement of phospholipids facilitates the transport of annexins A2 and A5 across membranes in cells and in liposomes. We identified TMEM16F (also known as anoctamin-6, ANO6) as a lipid scramblase required for transport of these annexins to the outer leaflet of the plasma membrane. This work reveals a mechanism for annexin translocation across membranes which depends on plasma membrane phospholipid remodelling.

## Abstract (precis)

**Summary:** Annexin secretion involves protein translocation across membrane that is facilitated by transbilayer lipid movement.

## INTRODUCTION

Most proteins are secreted via the endoplasmic reticulum (ER)/Golgi network. However, several cytosolic proteins are secreted by unconventional transport pathways (Nickel and Rabouille, 2009; Pompa et al., 2017). Decades of research has shown that protein families with members that use this pathway include annexins, glycolytic enzymes, heat shock proteins (HSPs), interleukins, fibroblast growth factors (FGFs), galectins, transglutaminases and misfolded proteins, among many others (Rabouille et al., 2012; Rabouille, 2017; Lee et al., 2016). This illustrates that unconventional secretion is a diverse and heterogeneous process. Over the years, the extracellular functions of these proteins have been well documented, as are associations of their perturbed secretion with several diseases (Gerke and Moss, 2002; Valapala et al., 2014; Zheng et al., 2011; Rabinovich et al., 2002; Chen et al., 2014; Vasta, 2009). However, the mechanism(s) of secretion and its regulation remain largely uncharacterised.

Multiple pathways have been proposed to explain the mechanisms of secretion and these can be divided into four main types (I to IV; Nickel and Rabouille, 2009; Rabouille et al., 2012; Rabouille, 2017). Interestingly, all four types of secretion involve crossing a membrane. For types I and II, proteins are directly translocated across the plasma membrane. In type I, protein translocation across the plasma membrane is either mediated by protein complexes, pores or is self-mediated (unfacilitated). In type II secretion, translocation is mediated by ATP-binding-cassette (ABC) transporter proteins. Type III describes the secretion of cytoplasmic proteins that first enter the lumen of an organelle, which then fuses with the plasma membrane. The type IV secretion concerns transmembrane proteins, which are inserted in the ER membrane but reach the plasma membrane after bypassing the Golgi.

Even though they do not share obvious common features in their sequence or structure, most unconventionally secreted proteins are reported to bind phospholipids (Lizarbe et al., 2013; van Genderen et al., 2008; Kobayashi et al., 1990; Schilling et al., 2009; Steringer et al., 2012; Zemskov et al., 2011). As such, we tested whether lipid binding and remodelling were important for direct protein translocation across membranes. In this study, we focused on annexins. Annexins are well-described Ca^2+^-dependent phospholipid-binding proteins. Annexins localise to the outer leaflet of the plasma membrane (cell surface) where they are involved in plasminogen activation leading to fibrinolysis and cell migration, among other functions (Gerke and Moss, 2002; Popa et al., 2018; Gerke et al., 2005). Despite having well-described extracellular functions, the mechanism of annexin transport from the cytoplasmic inner leaflet to the extracellular outer leaflet of the plasma membrane remains unclear. Annexins bind to negatively charged lipid head groups of the inner and outer leaflets of the plasma membrane in a Ca^2+^-dependent manner (Gerke and Moss, 2002; Kirsch et al., 1997); therefore, we hypothesised that lipid remodelling may be important for their translocation to the cell surface. Here, we show that the transbilayer movement of phospholipids is crucial for the transport of annexins A2 and A5 across membranes in cells and in liposomes. This mechanism is likely specific to annexins, as it is not shared by galectin-3, another unconventional cargo that binds phospholipids. Furthermore, we identified TMEM16F (also known as anoctamin-6, ANO6) as a lipid scramblase that facilitates the transport of these annexins to the outer leaflet of the plasma membrane. This work reveals a route for annexin translocation across membranes that depends on plasma membrane phospholipid movement.

## RESULTS

### Cinnamycin facilitates annexin translocation across membranes in cells

**Fig. 1.**
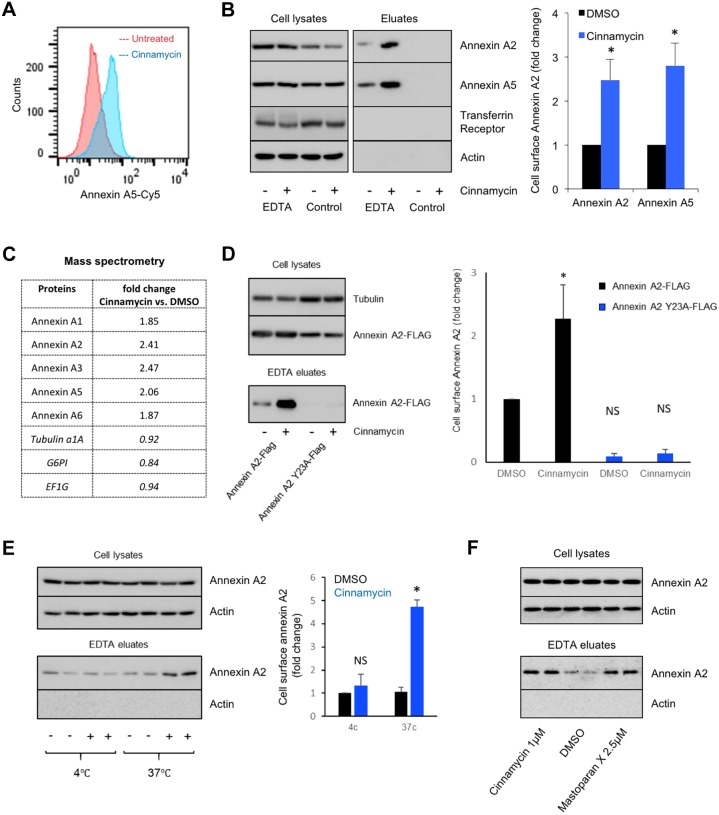
**Cinnamycin facilitates annexin translocation across membranes in cells.** (A) Cinnamycin lipid movement activity. HeLa cells were treated with 1 µM cinnamycin for 50 min at 37°C. Then, recombinant annexin-A5–Cy5 (as a probe for PS) and propidium iodide (PI) (to exclude PI-containing dead cells) were added, and cells were incubated for a further 10 min at 37°C. Annexin-A5–Cy5 binding and PI accumulation were analysed by flow cytometry. Representative histograms of annexin-A5–Cy5 binding to live cells are shown (*n*=3). (B) Western blotting analysis of cell lysates and eluates of HeLa cells treated with cinnamycin (30 min at 37°C) and then with EDTA (10 min at 37°C) as indicated. Quantification of cell surface annexin A2 and annexin A5 {fold change measured as band intensity [cinnamycin(eluate/lysate)/DMSO(eluate/lysate)]} is shown. Results are mean±s.e.m. from *n*=3 biological replicates; *P<0.05. (C) Mass spectrometry analysis of cell surface annexin from the samples in E. Data are fold change of the number of peptides identified measured as [cinnamycin(eluate/lysate)/DMSO(eluate/lysate)]. (D) Left, western blotting analysis of cell lysates and eluates of HeLa cells transfected for 24 h with annexin-A2–FLAG or annexin-A2-Y23A–FLAG, and then treated with cinnamycin (30 min at 37°C) and with EDTA (10 min at 37°C) as indicated. Right, quantification of cell surface annexin A2 {fold change measured as band intensity [cinnamycin(eluate/lysate)/DMSO(eluate/lysate)]}. Results are mean±s.e.m. from *n*=3 biological replicates; *P<0.05, NS: not significant. (E) Left, western blotting analysis of cell lysates and eluates of HeLa cells treated with cinnamycin (30 min at 37°C or 4°C) and then with EDTA (10 min at 37°C), as indicated. Right, quantification of cell surface annexin A2 {fold change measured as band intensity [cinnamycin(eluate/lysate)/DMSO(eluate/lysate)]}. Results are mean±s.e.m. from *n*=3 biological replicates; *P<0.05, NS: not significant. (F) Western blotting analysis of cell lysates and eluates of HeLa cells treated with cinnamycin or mastoparan X (1 h at 37°C) and then with EDTA (10 min at 37°C) as indicated.

To assess the role of lipid remodelling in annexin transport across membranes, we studied the effect of the lipid remodelling toxin cinnamycin in mammalian cells (Makino et al., 2003; Iwamoto et al., 2007). Cinnamycin is a 19-amino-acid lantibiotic that interacts with membranes by selectively recognising phosphatidylethanolamine (PE) and induces the movement of phospholipids including PE, phosphatidylserine (PS) and phosphatidylcholine (PC) from one leaflet of the lipid bilayer to the other, both in liposomes and cell membranes (Makino et al., 2003; Iwamoto et al., 2007). We confirmed that cinnamycin induces the transbilayer movement of PS and PE in HeLa cells by measuring the binding of recombinant annexin-A5–Cy5 bound to PE and PS on the cell surface by flow cytometry (Fig. 1A; Fig. S1A,B). Please note that recombinant annexin-A5–Cy5 is used as a read-out for PS/PE on the outer leaflet of the cell membrane and we do not measure annexin itself in the flow cytometry experiment. Next, we pre-treated HeLa cells with cinnamycin for 30 min in serum-free medium, washed the cells and dissociated all available cell surface annexin A2 and A5 with EDTA (Deora et al., 2004). Pre-treatment with cinnamycin dramatically increased the amount of annexin A2 and A5 in the EDTA eluate without impacting on cell morphology or viability, as shown through microscopy and measurement of the lactate dehydrogenase activity in the eluate fraction, respectively (Fig. 1B; Fig. S1C,D). As a negative control, no annexin A2 and A5 was detected in the eluate when cells were incubated in serum-free medium without EDTA (Fig. 1B). The pool of annexin A2 translocated from the cytosol to the cell surface in the presence of cinnamycin was in the range of 10–20% (Fig. S1E). The increase in annexin A2 and A5 on the cell surface in cinnamycin-treated cells was specific, as no cytosolic proteins or transmembrane proteins, such as actin, Arf1, Arf6 or the transferrin receptor, were detected in the eluate fractions (Fig. 1B; Fig. S1F). When cinnamycin was used at concentrations that compromised cell membrane integrity, we observed actin release in the eluate fraction (Fig. S1G), suggesting that actin in the eluate correlated with cell lysis. Mass spectrometry confirmed that cinnamycin stimulated translocation of annexin A1, A2, A3, A4 and A5 to the cell surface (Fig. 1C). This phenomenon was not limited to HeLa cells and could be demonstrated in several other lines (Fig. S1H).

It has previously been shown that a mutant of annexin A2 (Y23A), which is unable to bind to the membrane, is also defective in reaching the cell surface (Valapala et al., 2014; Zheng et al., 2011). To evaluate the importance of annexin A2 membrane binding in the translocation process, we analysed the transport of the annexin A2 mutant in the presence of cinnamycin. We observed that cinnamycin was unable to facilitate the translocation of this annexin A2 lipid-binding mutant (Fig. 1D). We also treated cells with cinnamycin at 4°C, where cinnamycin binds to the membrane but lipid movement is abrogated (Makino et al., 2003). We observed that cinnamycin-mediated annexin A2 translocation to the cell surface was inhibited at this temperature (Fig. 1E). Furthermore, mastoparan X, another toxin that causes lipid translocation (Matsuzaki et al., 1996), caused a similar increase in the amount of annexin A2 detectable on the cell surface (Fig. 1F). Taken together, these data support a mechanism whereby annexin A2 and A5 are transported to the cell surface by first binding to the inner leaflet of the membrane before being translocated across to the cell surface during lipid remodelling.

### Cinnamycin facilitates annexin translocation across membranes in liposomes

**Fig. 2.**
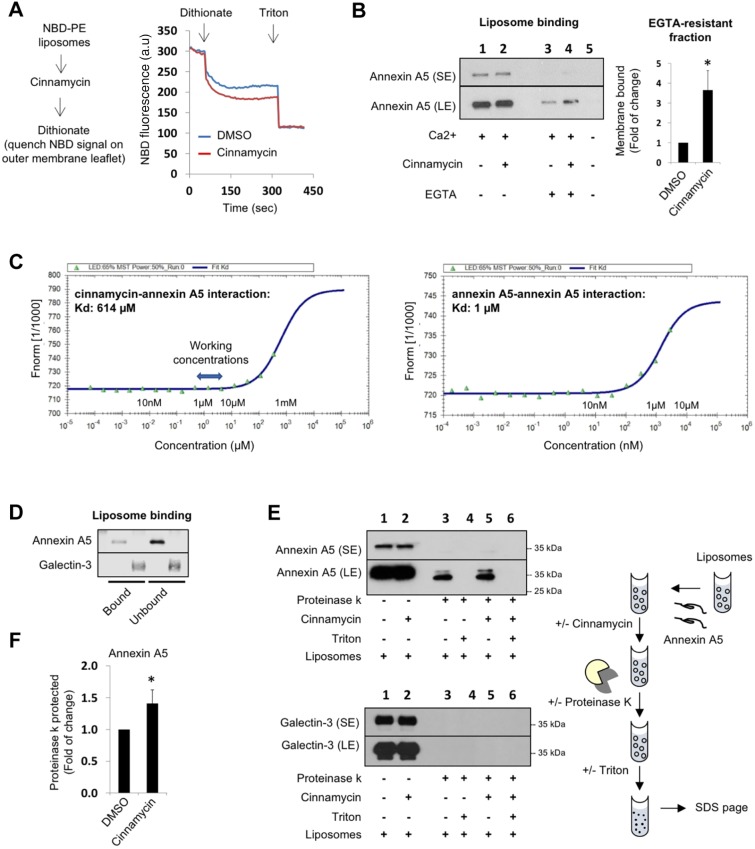
**Cinnamycin facilitates annexin translocation across membranes in liposomes but not galectin-3.** (A) Cinnamycin induces lipid movement in liposomes. Left, schematic of the lipid quenching assay. Right, LUVs (PC:PE 1:1, and a trace of NBD-PE) were pre-incubated with cinnamycin (10 µM) or DMSO for 30 min and changes in NBD fluorescence during the experiment time were recorded upon the addition of dithionite and Triton X-100. (B) Cinnamycin increases the EGTA-resistant membrane-bound fraction of annexin A5. PC:PE liposomes (MLVs) in CaCl_2_-containing buffer were incubated with annexin A5. Cinnamycin or DMSO was added for a further 40 min incubation at 37°C. Some of the samples were also treated with EGTA before all samples were centrifuged. Liposome pellets were mixed with boiling SDS sample buffer, separated by SDS-PAGE and analysed by western blotted with anti-annexin A5 antibodies for the detection of the membrane-bound fraction of annexin A5. Results are mean±s.d. comparing lane 3 vs. lane 4; *n*=3 independent experiments, *P<0.05. (C) Left, interaction between cinnamycin and annexin A5 as determined by microscale thermophoresis. The measurement gave a *K*_d_ of 614 µM. At the working concentration used in this study (1–10 µM), no interaction was obtained. Right, the dimerisation of annexin A5 in the presence of Ca^2+^ was used as a positive control for the assay. A *K*_d_ of 1 µM was obtained. (D) Recombinant annexin A5 or galectin-3 were incubated with PC:PE liposomes (MLVs, 1 mM) in CaCl_2_-containing buffer for 30 min before samples were centrifuged. Liposome pellet and supernatant were resolved in SDS-PAGE and probed with anti-annexin A5 and anti-galectin-3 antibodies. (E) Protease protection assay (western blot). PC:PE liposomes (LUV) in CaCl_2_ containing buffer were incubated with FITC-Annexin A5 or galectin-3 recombinant proteins. Cinnamycin or DMSO was added for further 40 min incubation at 37°C. Proteinase K alone or together with Triton X-100 (0.5%) was added to liposome samples for further 1 h at 37°C. After protease inactivation and boiling with SDS sample buffer, samples were resolved by SDS-PAGE and the proteinase K-protected fraction of Annexin A5 and galectin-3 was detected by western blot analysis with anti-annexin A5 and anti-galectin-3 antibodies. A schematic of the experiment is shown on the right. (F) Quantification of the proteinase K-protected fraction from E. Results are mean±s.d. comparing lane 3 versus lane 5, *n*=4 independent experiments, *P<0.05. SE, short exposure; LE, long exposure.

To evaluate whether the movement of lipids is the minimal requirement for annexin transport across membranes, we developed an *in vitro* liposome system using recombinant annexin A5 and cinnamycin. We first confirmed that cinnamycin could induce lipid movement in liposomes using an established assay based on the quenching of NBD–PE in the outer membrane leaflet with dithionite (Fig. 2A) (Menon et al., 2011). Next, we performed liposome binding and sedimentation experiments that showed that recombinant annexin A5 binds to phosphatidylcholine:phosphatidylethanolamine (PC:PE) liposomes in the presence of Ca^2+^ and that the majority of bound material is removed from the membrane upon treatment with the Ca^2+^ chelator EGTA (Fig. 2B, lane 1 versus 3). We reasoned that if cinnamycin can lead to the translocation of annexins *in vivo*, we could mimic this in liposomes, with cinnamycin treatment leading to an increase in the fraction of annexin A5 protected inside the lumen of liposomes. In the absence of cinnamycin, a small fraction of annexin A5 was EGTA resistant (Fig. 2B, lane 3) suggesting that it was either inserted into membranes or protected in the lumen of the liposomes. This is in agreement with previous liposome studies on the role of annexin A5 as a mediator of Ca^2+^ flux across membranes (Kirsch et al., 1997). Pre-treatment with cinnamycin increased the EGTA-resistant annexin A5 fraction (Fig. 2B, lane 4), suggesting that annexin A5 is translocated from the outer leaflet of the liposome membrane (surface of the liposome) to the inner leaflet or lumen of the liposome, where it was protected from removal by EGTA. The translocation of annexin A2 upon cinnamycin treatment was not mediated by the direct interaction between the two proteins as no interaction could be detected in a microscale thermophoresis assay at the concentration of cinnamycin used in our assay (10 μM; Fig. 2C). We could only detect an interaction between cinnamycin and annexin A5 when using cinnamycin at 1 mM, which is a very low affinity interaction. As a positive control for the microscale thermophoresis assay, we measured the dimerisation of annexin A5 in the presence of Ca^2+^, as previously reported in ref (Neumann et al., 1994), and obtained a *K*_d_ of 1 μM (Fig. 2C).

To confirm the translocation of annexin A5 across membranes, we performed a proteinase K protection assay. Proteinase K is very efficient in cleaving exposed proteins from membrane surfaces. We also used recombinant galectin-3, another unconventionally secreted phospholipid-binding protein (Lukyanov et al., 2005), in our experiment to assess whether the translocation was specific to annexins. Using a sedimentation assay, we confirmed that both annexin A5 and galectin-3 bind PC:PE liposomes (Fig. 2D). Next, PC:PE liposomes were pre-incubated with either annexin A5 or galectin-3 before incubation with proteinase K. Proteinase K treatment resulted in digestion of free and surface-bound annexin A5, as detected through a dramatic decrease in amount of full-length annexin A5 seen by western blot (Fig. 2E, lane 1 versus 3). A small fraction of full-length annexin A5 and a near full-length cleavage product was also detected; these corresponded to fully protected and partially membrane-inserted annexin A5, respectively (Fig. 2E, top panel lane 3). In contrast, galectin-3 was fully digested by the protease, suggesting that it was not inserted into the bilayer of the liposomes (Fig. 2E, bottom panel lane 3). The fraction of annexin A5 protected from protease cleavage was membrane dependent, as all available annexin A5 was degraded in the presence of membrane-solubilising detergent (Triton X-100) (Fig. 2E, top panel lane 4). Importantly, cinnamycin pre-treatment increased the levels of the full-length annexin A5 and the near full-length cleavage product detectable after proteinase K digestion (Fig. 2E, top panel lane 5 and Fig. 2F). Therefore, cinnamycin increased the proportion of annexin A5 protected from proteinase K. The relative increase in the membrane-translocated fraction of annexin A5 measured during the protease assay (Fig. 2F) is smaller than that seen in the EGTA assay (Fig. 2B). The protease assay requires a fast inactivation and denaturation of the proteinase K prior to sample analysis by SDS-PAGE to avoid further cleavage of annexin A5. Owing to this limitation, it is possible that not all of the proteinase K was efficiently inactivated, resulting in a lower level of protected annexin A5. Nevertheless, we observed the same trend following cinnamycin treatment in both the EGTA and the protease assays. Notably, cinnamycin was unable to protect galectin-3 from complete degradation (Fig. 2E, bottom western blot lane 5, Fig. 2F), suggesting that protein insertion into the membranes is a requirement for the protein translocation process to occur.

**Fig. 3.**
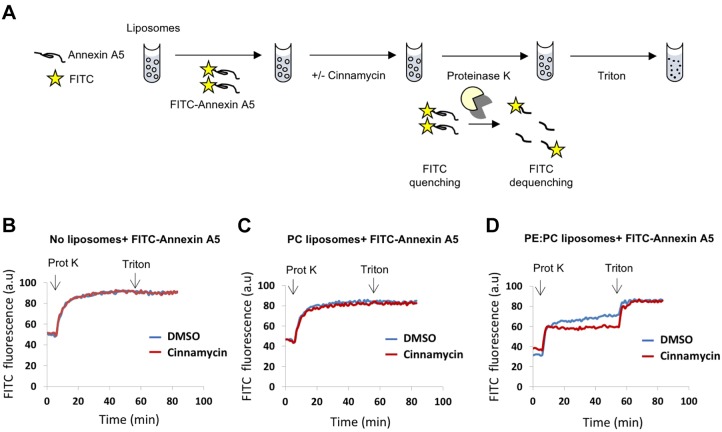
**Effect of different phospholipids on cinnamycin-mediated annexin translocation across membranes.** (A) Schematic of the proteinase K-mediated FITC-annexin A5 dequenching assay. (B–D) Protease protection assay analysing proteinase K-induced FITC–annexin-A5 dequenching in liposomes with different phospholipid compositions. FITC–annexin-A5 was added to buffer alone (B), a PC-only LUV suspension (C) or a PE:PC LUV suspension (D). Cinnamycin or DMSO was added to the samples and changes in FITC fluorescence during the experiment time were recorded at 37°C. Proteinase K was added to the samples followed by addition of Triton X-100 (0.5%). Results are average of duplicate measurements. Similar effects were detected in two independent experiments.

A similar protection assay was used in combination with a fluorescence-quenching assay, allowing a real-time readout for annexin A5 degradation (Fig. 3A). In this assay, recombinant annexin A5 was labelled with fluorescein isothiocyanate (FITC) molecules. At high density, the fluorescence of the FITC molecules is quenched owing to their close proximity on annexin A5. When proteinase K is added, accessible FITC–annexin-A5 is cleaved, allowing the FITC molecules to be spatially separated, triggering an increase in their intrinsic fluorescence (dequenching) (Fig. 3A). Proteinase K-induced FITC–annexin-A5 dequenching was unaffected when incubated with cinnamycin then Triton X-100 without liposomes, or when incubated with PC-only liposomes (Fig. 3B,C). This is expected as neither annexin A5 nor cinnamycin binds PC membranes (Choung et al., 1988). By contrast, proteinase K-induced FITC-annexin A5 dequenching was reduced when PE-containing liposomes were used (Fig. 3D), consistent with our results from the previous protection assay, indicating that there is a fraction of annexin A5 that is protected from protease cleavage (Fig. 2E). Furthermore, in agreement with previous results, PE-containing liposomes pre-treated with cinnamycin exhibited a higher degree of attenuated FITC dequenching, compared to the control (DMSO treatment) (Fig. 3D). Finally, the addition of Triton X-100, which solubilises the membranes, led to a complete recovery in FITC fluorescence in both the DMSO and cinnamycin conditions (Fig. 3D). Interestingly, cinnamycin induced dequenching of annexin A5 in the presence of PE liposomes (Fig. 3D, 0–5 min). Reasons for this may include: (1) that cinnamycin competes with pre-bound annexin A5 on liposomes leading to a lower amount of annexin A5 binding to each liposome, or (2) that cinnamycin could lead to annexin translocation into the liposome lumen. However, we are unable to distinguish between these possibilities. Taken together, these three approaches suggest that the transbilayer movement of phospholipids induced by cinnamycin facilitates annexin A5 translocation across liposome membranes in a dynamic process independent of energy sources, such as ATP.

### TMEM16F is required for annexin localisation on the cell surface

**Fig. 4.**
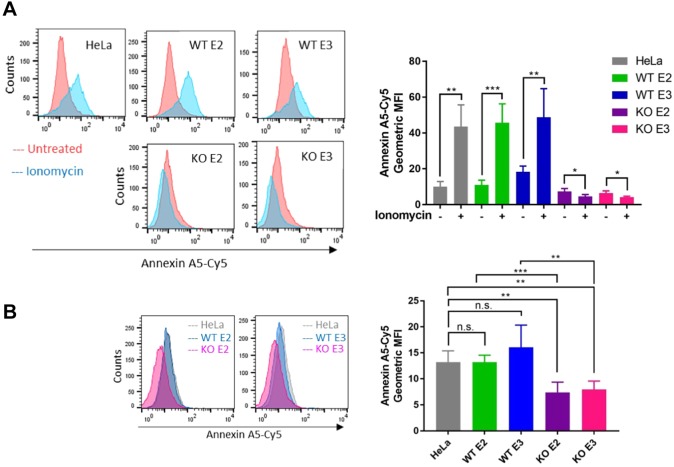
**TMEM16F regulates lipid movement in HeLa cells.** (A) TMEM16F-knockout (KO) cells do not externalise PS in response to ionomycin stimulation. Wild-type (WT), matched controls and TMEM16F-knockout cells were treated with ionomycin for 10 min at 37°C in the presence of recombinant annexin-A5–Cy5 and PI. Recombinant annexin-A5–Cy5 binding and PI accumulation were analysed by flow cytometry. Representative histograms of recombinant annexin-A5–Cy5 binding to live cells are shown (*n*=4). A quantification of the geometric mean±s.d. fluorescence intensity of annexin-A5–Cy5 binding from four separate experiments are shown. *P<0.05, **P<0.01, ***P<0.001. (B) Basal cell surface PS is reduced in TMEM16F-knockout cells. Cells were incubated with recombinant annexin-A5–Cy5 and PI for 10 min at 37°C before flow cytometry analysis. Representative histograms are displayed (*n*=5). The geometric mean±s.d. fluorescence intensity of annexin A5-Cy5 is plotted from five separate experiments. E2, exon 2 targeted; E3, exon 3 targeted. **P<0.01, ***P<0.001, n.s., not significant.

Given that cinnamycin-induced lipid movement is sufficient to translocate annexins across membranes in cells and liposomes, we looked for a mammalian protein that could drive this process in cells. Plasma membrane lipid asymmetry is maintained by transmembrane proteins that move lipids from the inner leaflet to the outer leaflet and vice versa (Daleke, 2003; Pomorski and Menon, 2016). One family of proteins with lipid translocation activity are the scramblases (Sahu et al., 2007). Scramblase activity is Ca^2+^ dependent and energy independent (Pomorski and Menon, 2016; Suzuki et al., 2010). Scramblase dysfunction results in Scott's syndrome, a mild bleeding disorder thought to result from a lack of PS externalisation (Zwaal et al., 2004). Scott's syndrome has been attributed to mutations in the phospholipid scramblase known as TMEM16F (Suzuki et al., 2010). Owing to the importance of lipid movement activity of TMEM16F *in vivo*, we investigated a role for TMEM16F in the translocation of annexin A2 and A5 to the cell surface. Clonal TMEM16F HeLa-knockout cell lines were generated using CRISPR/Cas9 and matched wild-type controls with no targeting were also isolated (Fig. S2A,B). We confirmed gene targeting by performing sequencing and quantitative real-time PCR (qPCR) (Fig. S2C). We were unable to measure the protein level of TMEM16F as the few antibodies that we tried did not show specific signals on western blots (data not shown). However, we confirmed a functional defect in lipid translocation in the TMEM16F-knockout cell lines by measuring the level of PS on the cell surface in cells challenged with ionomycin, a Ca^2+^ ionophore that stimulates PS externalisation (Fig. 4A; Fig. S3A) (Zwaal et al., 2005). We treated TMEM16F wild-type or -deficient cells with ionomycin for 10 min at 37°C in the presence of recombinant annexin-A5–Cy5 and propidium iodide. Cell surface PS (and PE) were analysed by measuring the amount of recombinant annexin-A5–Cy5 bound to live cells by flow cytometry. As above, please note that recombinant annexin-A5–Cy5 is used as a read-out for PS/PE on the outer leaflet of the cell membrane, and we do not measure annexin itself by flow cytometry in any assay. TMEM16F-deficient cells were unable to externalise PS in response to an increase in intracellular Ca^2+^, whereas wild-type cells and positive matched non-targeted controls showed an increase in the amount of PS on the cell surface (Fig. 4A; Fig. S3A). These data confirm that TMEM16F activity is abolished in TMEM16F-knockout cells. Interestingly, the level of PS (and PE) on the cell surface of TMEM16F-deficient cells under unstimulated conditions was also slightly reduced compared to the wild-type and untargeted controls (Fig. 4B), showing for the first time that TMEM16F is active under basal conditions. Furthermore, there is a small and consistent decrease in the amount of PS on the cell surface of TMEM16F-deficient cells when challenged with ionomycin. We hypothesise that this is due to the ionomycin-stimulated activity of a flippase that translocates lipids from the outer leaflet of the plasma membrane to the inner leaflet.

**Fig. 5.**
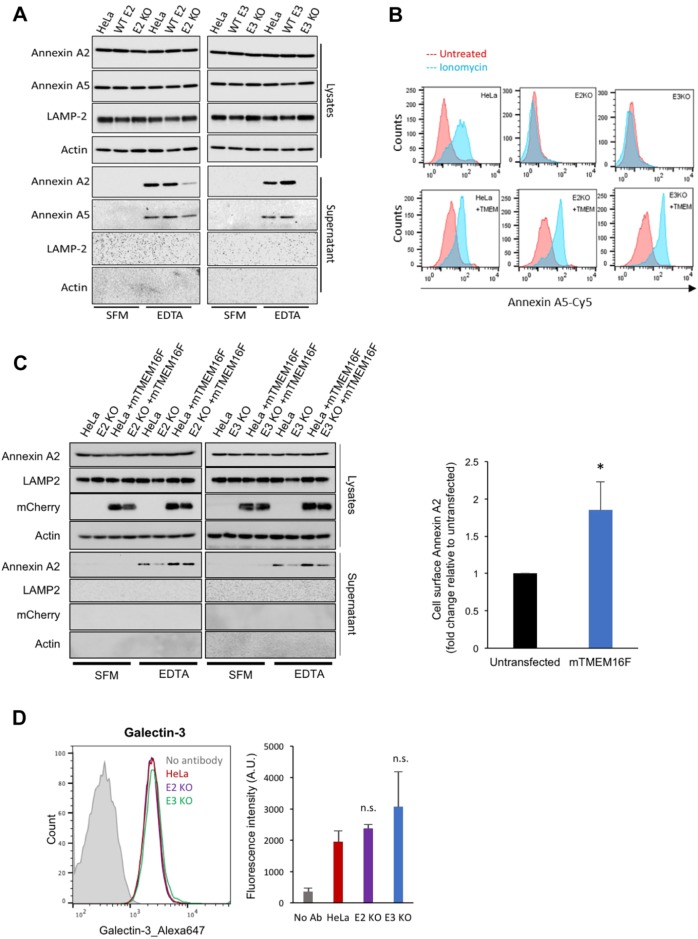
**TMEM16F is required for annexin A2 and A5 cell surface localisation.** (A) TMEM16F-knockout (KO) cells have severely reduced annexin A2 and A5 on their surface. Wild-type (WT), matched controls and TMEM-knockout HeLa cells were incubated in versene (EDTA solution) or not (SFM) for 10 min at 37°C before the eluate was collected and analysed for annexin A2 and A5 by western blotting. A representative western blot is shown (*n*=4). (B) Expression of mCherry–mTMEM16F rescues lipid movement in TMEM16F-knockout cells. Wild-type and TMEM16F-knockout HeLa cells alone or expressing mCherry–mTMEM16F were treated with ionomycin and analysed for recombinant annexin-A5–Cy5 binding by flow cytometry. A representative experiment is shown (*n*=3). (C) Expression of mCherry–mTMEM16F rescues annexin A2 and A5 expression at the cell surface. Annexin A2 and A5 on the cell surface were evaluated through treatment with EDTA and western blotting as described in A. A representative experiment is shown (*n*=4). A quantification of cell surface annexin A2 in HeLa transfected or not with mTMEM16F is presented (fold change measured as band intensity [transfected(eluate/lysate)/untransfected(eluate/lysate)]) Results are mean±s.e.m. from *n*=5 biological replicates; *P<0.05. (D) Cell surface localisation of galectin-3 in the TMEM16F-knockout cells. Cells were incubated with an anti-galectin-3 antibody conjugated with Alexa Fluor 647 for 30 min at 4°C. Cells were washed and processed for flow cytometry. The geometric mean fluorescence intensity of galectin-3–Alexa-Fluor-647 is shown obtained from three separate experiments. E2, exon 2 targeted; E3, exon 3 targeted. n.s., not significant.

To investigate the role of TMEM16F in translocation of annexins, we assessed the level of annexin A2 and A5 on the cell surface of TMEM16F-deficient cells. Cells were treated with EDTA to release annexin A2 and A5, and the eluate was assessed by western blotting, as described above. Strikingly, in both TMEM16F-deficient clones, the level of annexin A2 and A5 were severely reduced in the EDTA eluate (Fig. 5A). Annexin A2 and A5 were not detected in the serum-free medium (SFM) eluate, and actin and LAMP-2 were absent from all eluates (SFM or EDTA), indicating that this was a specific process (Fig. 5A). To ensure that the lack of annexin on the cell surface was due to reduced translocation rather than reduced retention at the cell surface, we assessed the level of annexin released into the supernatant over 24 h. No annexin A2 or A5 was detected in the supernatant from wild-type or TMEM16F-deficient cells (Fig. S3B). This demonstrated that annexin A2 and A5 were not translocated, and thus not present, in the medium in TMEM16F-deficient cells (Fig. S3B), whereas annexin A2 and A5 are translocated across the membrane in wild-type cells and are detected on the cell surface (Fig. 5A). The lack of annexin A2 and A5 on the cell surface in TMEM16F-deficient cells is not due to off-target effects, as the phenotype is consistent across both clones targeted with different sgRNAs. Furthermore, the phenotype was normalised when the TMEM16F-deficient cells were reconstituted with mouse mCherry-tagged TMEM16F (mCherry–mTMEM16F) via lentiviral transduction, which restored lipid movement activity (Fig. 5B,C; Fig. S4A–D). The overexpression of mCherry–mTMEM16F by itself was also sufficient to increase the amount of annexin on the cell surface in wild-type cells (Fig. 5C), further supporting a role for TMEM16F in the secretion of annexin. In accordance with our *in vitro* data on galectin-3, TMEM16F-knockout cells did not show any defect in the localisation of galectin-3 to the cell surface (Fig. 5D). This established that TMEM16F is specifically required for the transport of annexin to the cell surface.

**Fig. 6.**
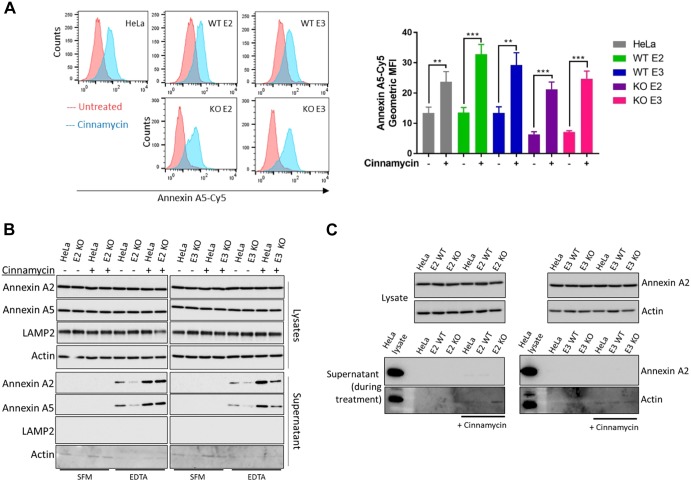
**Cinnamycin restores annexin A2 and A5 cell surface localisation in TMEM16F-deficient cells.** (A) Cinnamycin externalises PS in TMEM16F-knockout (KO) cells. Wild-type (WT), matched controls and TMEM16F-knockout cells were treated with cinnamycin for 50 min at 37°C, recombinant annexin-A5–Cy5 and PI were then added and incubated for a further 10 min at 37°C. Recombinant annexin-A5–Cy5 binding and PI accumulation were analysed by flow cytometry. Representative histograms of recombinant annexin-A5–Cy5 binding to live cells are shown (*n*=3). The geometric mean±s.d. fluorescence intensity of annexin A5-Cy5 binding to live cells are shown from three separate experiments. **P<0.01, ***P<0.001. (B) Annexin A2 and A5 cell surface localisation is restored after cinnamycin treatment. Wild-type, positive control and TMEM-knockout HeLa cells were incubated with DMSO or cinnamycin at 37°C for 1 h. Cells were washed and annexin A2 and A5 released with versene (EDTA solution) or not (SFM) for 10 min at 37°C before the eluate was collected and analysed through western blotting. A representative western blot is shown (*n*=4). (C) Annexin A2 and A5 are retained on the cell surface and not secreted in the presence of cinnamycin. The medium was collected after 1 h of cinnamycin treatment and analysed for annexin A2 and A5 free in the medium. Actin is used as a control. E2, exon 2 targeted; E3, exon 3 targeted.

Our data indicate that cinnamycin stimulates lipid movement in a manner comparable to that mediated by TMEM16F; therefore, we set out to determine whether its activity could substitute for TMEM16F. Cells were treated with cinnamycin, and the level of PS and PE externalisation was determined by assessing the binding of recombinant annexin-A5–Cy5 to live cells by flow cytometry. Cinnamycin stimulated lipid externalisation in both wild-type and TMEM16F-deficient cells (Fig. 6A; Fig. S5). Therefore, cinnamycin can be used as a surrogate for the TMEM16F lipid movement activity. TMEM16F-deficient cells treated with DMSO showed reduced annexin A2 and A5 in the EDTA eluate (Fig. 6B); however, when treated with cinnamycin there was a significant increase in the amounts of annexin A2 and A5 detectable in the EDTA eluate in these cells (Fig. 6B). Cinnamycin also increased the level of annexin A2 and A5 on the cell surface in wild-type HeLa cells, as expected (Fig. 6B). This demonstrates that lipid remodelling activity is sufficient for translocation of annexin A2 and A5 from the cytosol to the cell surface and that this was not due to a lack of annexin retention at the cell surface as no significant amount of annexin A2 was present in the medium during cinnamycin treatment as described above (Fig. 6C).

## DISCUSSION

Many proteins with important extracellular functions are known to be secreted through an unconventional pathway; however, the details of this process remain elusive. Using both liposomes and mammalian cells, we show that lipid remodelling facilitates translocation of annexin A2 and A5 across membranes. Our liposome data indicate that this translocation is ATP independent and does not require the complex machinery seen for conventional trafficking of proteins into the ER (Nickel and Rabouille, 2009; Pompa et al., 2017). In cells, we confirm that TMEM16F mediates PS and PE externalisation and lipid remodelling, and show that this occurs both in the steady state and during ionomycin stimulation. We further add to this, demonstrating that TMEM16F expression is also required for localisation of annexin A2 and A5 on the cell surface. In TMEM16F-deficient cells, surface localisation of annexin A2 and A5 can be restored by reconstituting TMEM16F expression or by stimulating transbilayer lipid movement with cinnamycin. This provides important insights into the unconventional secretion of annexins, highlighting a previously unknown role for lipid remodelling in the translocation process. Additionally, we describe a new function for the phospholipid scramblase TMEM16F in facilitating unconventional secretion of annexins.

We also investigated whether lipid remodelling by TMEM16F was a general regulator of unconventional secretion by studying the secretion of galectin-3. We found that galectin-3 cell surface localisation is not impaired by the absence of TMEM16F. Furthermore, unlike what was seen for annexin A5, when liposomes were treated with cinnamycin, no galectin-3 was translocated into the lumen. Therefore, it is likely that there is a separate and distinct pathway for the unconventional secretion of galectins.

Another unconventional cargo that would fit with this requirement is fibroblast growth factor 2 (FGF2) (Steringer et al., 2017; Steringer and Nickel, 2018). FGF2 is able to bind to the inner leaflet of the plasma membrane in a lipid-dependent manner and then self-assembles into the membrane forming a pore-like structure (Steringer et al., 2017). FGF2 is finally pulled through the membrane via an electrostatic exchange from the inner leaflet of the membrane onto the outer leaflet (cell surface) (Steringer et al., 2017; Steringer and Nickel, 2018). Therefore, it is reasonable to speculate that the transbilayer lipid movement induced by cinnamycin or TMEM16F could stimulate FGF2 translocation across membrane, similar to what is seen for the annexins.

Here, we propose a mechanism whereby transbilayer lipid movement facilitates annexin translocation, a process that can be mediated by either cinnamycin or TMEM16F. In a broad sense cinnamycin and TMEM16F act in a similar way; however, the differences in how they mediate lipid translocation is unclear. It is thought that TMEM16F is a large transmembrane protein that mediates lipid translocation by conducting lipid head groups through its hydrophilic groove, which spans the plasma membrane (Bethel and Grabe, 2016). Cinnamycin on the other hand is a much smaller peptide that binds to PE and causes transbilayer movement accompanied by a structural change in the peptide. Therefore, it appears that general transbilayer lipid movement is the major requirement for annexin translocation across the membrane.

It has been reported that annexins bind to the membrane in a cooperative manner, which means either annexin monomers create a higher affinity binding site for another by self-rearrangement or by rearrangement of the membrane (Drucker et al., 2014). It remains unclear whether annexin A2 oligomerises or forms a heterotetrameric complex prior to secretion. The cooperative nature of the binding of annexin A2 monomers to supported lipid bilayers suggests that annexin A2 binds in clusters on the plasma membrane. This could be important for their transbilayer-mediated movement to the cell surface. If TMEM16F is facilitating the movement of PS from the inner leaflet to the outer leaflet of the membrane during scrambling, perhaps annexin A2 bound to phospholipids on the inner leaflet would follow, either remaining bound to the lipid head groups or following the increased concentration of PS on the outer leaflet (similar to FGF2). This would work particularly well in lipid raft-like situations where there is a locally high concentration of phospholipids, PI(4,5)P_2_ and cholesterol, where several annexin A2 molecules are bound. As the phospholipids translocate, annexin A2 molecules may follow given that they may already be partially inserted into the membrane and they have a high affinity for specific phospholipid head groups.

Most reports on unconventional secretion of annexins have studied cells during stress or after interferon-γ stimulation (Chen et al., 2017; Fang et al., 2012). It has been shown that more annexin A2 is secreted in response to interferon-γ stimulation, and that this is mediated by extracellular vesicles (Chen et al., 2017). In contrast, we have investigated annexin secretion and subsequent retention at the cell surface under unstimulated (‘healthy’) conditions. We also find that annexin A2 and A5 localise to the cell surface rather than being secreted into the medium, suggesting that under resting conditions very little annexin A2 and A5 is free or present in extracellular vesicles.

Finally, our data are consistent with the physiological roles of extracellular annexins and TMEM16F, and highlight a potential fine balance required for maintaining homeostasis. It has been shown that cell surface annexin positively regulates cell migration and TMEM16F-deficient cells show a reduction in cell migration (Jacobsen et al., 2013); therefore, this defect could be explained by the loss of annexin on cell surface. Annexins have long been proposed to play an anti-thrombotic role on the cell surface (Reutelingsperger et al., 1985; Hauptmann et al., 1989). TMEM16F-knockout mice show increased bleeding whereas annexin A2-knockout mice show increased thrombosis and fibrin accumulation (Ling et al., 2004; Fujii et al., 2015). Annexin A5 has also been suggested to regulate thrombosis (Ida et al., 2004). These are opposing effects, which suggest that this pathway is a tightly regulated process that requires both TMEM16F activity and annexins on the cell surface. If injury is sufficient, PS externalisation overcomes the regulation by annexins and coagulation proceeds. If not, thrombosis is controlled by annexins. This fine balance is very interesting, and requires further investigation at steady state and during blood clotting.

In conclusion, we have described insights into the unconventional secretion of annexins, whereby annexin A2 and A5 translocation is facilitated by lipid reorganisation mediated by the phospholipid scramblase TMEM16F. Future work is needed to fully appreciate the importance and implications of this unconventional secretory pathway for lipid-binding cytosolic proteins.

## MATERIALS AND METHODS

### Cell culture

HeLa cells (from the ATCC) were cultured in DMEM (D6546, Molecular Probes) containing 10% fetal bovine serum, supplemented with 2 mM L-glutamine and 100 U ml^−1^ penicillin/streptomycin in 5% CO_2_ at 37°C. PANC-1 and AsPC-1 (a gift from Frances Richards, Cancer Research UK, Cambridge Institute, UK) were cultured in DMEM (D6546, Molecular Probes) containing 10% fetal bovine serum, supplemented with 2 mM L-glutamine and 100 Uml^−1^ penicillin/streptomycin in 5% CO_2_ at 37°C, or RPMI-1640 (Molecular Probes) containing 10% fetal bovine serum, supplemented with 2 mM L-glutamine and 100 Uml^−1^ penicillin/streptomycin in 5% CO_2_ at 37°C, respectively. Human embryonic kidney (HEK) 293 cells were cultured in DMEM (D6546, Molecular Probes) containing 10% fetal bovine serum, supplemented with 2 mM L-glutamine and 100 U ml^−1^ penicillin/streptomycin in 5% CO_2_ at 37°C. All cells tested negative for mycoplasma contamination.

### Antibodies

Antibodies used were: mouse monoclonal anti-annexin A2 (BD Biosciences; 610071; 1:1000), mouse monoclonal anti-annexin A5 (Abcam; ab54775, 1:1000), mouse monoclonal anti-transferrin receptor (Zymed; H68.4; 1:1000), rabbit polyclonal anti-actin (Sigma; A2066; 1:2000), mouse monoclonal anti-tubulin (Sigma; T9026; 1:4000), mouse monoclonal anti-FLAG (Sigma-Aldrich; clone M2; 1:4000), mouse monoclonal anti-Arf1 (Santa Cruz Biotechnology; sc-53168, 1:1000), rat anti-galectin-3 (Biolegend; 125401; western blotting, 1:2000) mouse monoclonal anti-Arf6 (Santa Cruz Biotechnology; clone 3A-1; 1:500), mouse monoclonal anti-LAMP2 (Biolegend; 354302; western blotting, 1:1000), rat polyclonal anti-galectin-3 conjugated to Alexa Fluor 647 (Biolegend; 125408; flow cytometry, 1:100) and rabbit polyclonal anti-mCherry antibody (Genetex; GTX128508-S).

### Reagents

Reagents used were: cinnamycin (Santa Cruz Biotechnology; sc-391464), mastoparan X (Alfa Aesar; J61173), recombinant annexin-A5–FITC (Abcam; ab14085), 1,2-dioleoyl-sn-glycero-3-phosphoethanolamine (DOPE, Sigma-Aldrich; 54008), 2-oleoyl-1-palmitoyl-sn-glycero-3-phosphocholine (POPC, Sigma-Aldrich; P3017), 1,2-dioleoyl-sn-glycero-3-phosphoethanolamine-N-(7-nitro-2-1,3-benzoxadiazol-4-yl) (NBD-PE, Avanti Polar Lipids, 810145) and proteinase K (Molecular Biology; BP1700-100), EGTA (Sigma-Aldrich; E3889), sodium dithionite (Sigma-Aldrich; 71699), versene solution containing ethylenediaminetetraacetic acid (EDTA) (Gibco; 15040-033), propidium iodide solution (Biolegend; 421301), QuickExtract DNA extraction solution (Epicenter; QE0905T), Herculase II fusion DNA polymerase (Agilent; 600675), annexin-V–FITC and annexin-V–Cy5 Apoptosis Staining/Detection Kit (ab14085, ab14150), annexin-V conjugated to Alexa Fluor 647 (Biolegend; 640912) and ionomycin (Cayman Chemical Company; 10004974), recombinant galectin-3 (Biolegend; 599706) and recombinant anneaxin A5 (Novus NBP1-30265). Oligonucleotides for TMEM16F CRISPR targeting and sequencing were synthesised from Sigma-Aldrich (Table S1).

### Plasmids

Annexin A2-FLAG and Annexin A2 Y23A-FLAG were a gift from Lei Zheng (Johns Hopkins Technology Ventures, Baltimore, MD) (Zheng et al., 2011), pSpCas9(BB)-2A-Puro (PX459) was Addgene plasmid #48139 (deposited by a gift from Feng Zhang; Ran et al., 2013). ANO6-Plvx-mCherry-c1 was Addgene plasmid #62554 (deposited by Renzhi Han; Zhao et al., 2014). psPAX2 and pMD2.G were Addgene plasmid #12260 and #12259, respectively (deposited by Didier Trono).

### Preparation of liposomes

Thin films were generated following dissolution of the lipids in a 2:1 (v/v) chloroform/methanol mixture and then dried under a stream of argon gas while they were rotated. The final compositions in mole percentage of the different liposome population were: PE-containing liposomes, 50% DOPE and 50% POPC; and PC-containing liposomes, 100% POPC. The films were lyophilised overnight, and the containers were sealed with argon gas to prevent oxidation and stored at −20°C. Multilamellar vesicles (MLVs) were generated by solubilising the lipid films with physiologic salt buffer (PSB), composed of 100 mM KOAc, 2 mM Mg(OAc)_2_ and 50 mM HEPES pH 7.4, using vigorous vortexing. For generation of large unilamellar vesicles (LUVs), the films were suspended in PSB and vortexed for 1.5 min. The lipid suspension underwent five cycles of freezing and thawing followed by extrusion through polycarbonate membranes with 1 and 0.1 μm diameter pores (from Avanti Polar Lipids) to create LUVs, as previously described (Vicinanza et al., 2015).

### Measuring lipid movement in liposomes

The lipid movement assay in liposomes was undertaken using a well-established procedure (Menon et al., 2011). Briefly, fluorescence measurements were performed using a SpectraMax M5 (Molecular Devices) in 96-well plates with total reaction volumes of 100 µl at a constant temperature of 37^°^C. Excitation was set on 480 nm, and emission was set on 530 nm (NBD fluorescence) with low photomultiplier tube (PMT) sensitivity. LUVs (PC:PE 1:1, and 0.6% NBD-PE) at 500 µM in PSB were pre-incubated with cinnamycin (10 µM) or DMSO, as a control, for 30 min, and changes in NBD fluorescence during the experimental period were recorded and the effects of dithionite (3 mM) were assessed. Dithionite reduces the NBD molecules on the head group of the lipids. Because only NBD in the outer membrane leaflet is accessible to react with dithionite, a partial decrease in fluorescence is observed. When the fluorescent system reached a steady-state, a membrane-solubilising detergent (0.05% Triton X-100) was added, which exposes the NBD in the inner leaflet and the decrease in NBD fluorescence was assessed. If cinnamycin induces transbilayer lipid movement, then there would be a difference in the level of fluorescence (compared to DMSO control) after dithionite treatment, which correlates to the different level of NBD–PE in the outer leaflet.

### Annexin A5 liposome-binding experiments

An MLV suspension (1 mM) was supplemented with CaCl_2_ (5 mM) and was incubated with FITC–annexin-A5 (1:7 dilution from stock solution) for 30 min in room temperature to allow annexin A5 binding to liposomes. Cinnamycin (10 µM) or DMSO was added for a further 40 min incubation at 37°C. Some of the samples were also treated with EGTA (10 mM) for 20 min in in room temperature before all samples were centrifuged (16,000 ***g***, 30 min at 4°C). Liposome pellets were mixed with boiling SDS-sample buffer for 1 min, separated by SDS-PAGE, transferred onto PVDF membranes and subjected to western blot analysis with anti-annexin A5 antibodies for the detection of the membrane-bound fraction of annexin A5.

### Protease protection assay

#### SDS-PAGE analysis

A LUV suspension (100 µM) was supplemented with CaCl_2_ (5 mM) and incubated with recombinant FITC–annexin-A5 (1:4.5 dilution from stock solution) or recombinant galectin-3 for 30 min at room temperature. Cinnamycin (10 µM) or DMSO was added for a further 40 min incubation at 37°C. For proteinase K experiments, a stock solution of the protease was freshly prepared by in PSB (10 mg/ml) prior to the experiment and was kept on ice. Proteinase K was added to liposome samples (1:20 dilution from stock solution) and incubation was carried out for a further 1 h at 37°C to allow full digestion. For some samples, Triton X-100 (0.5%) was added together with Proteinase K. We then inactivated the protease by adding a small amount of PMSF (from 0.25 M stock dissolved in DMSO) to the liposome samples, and put this on ice. The proteolysis samples were transferred into boiling SDS sample buffer and immediately pipetted several times. The boiled protein samples were resolved by SDS-PAGE and the proteinase K-protected fraction of annexin A5 and galecin-3 was detected by western blot analysis with anti-annexin A5 and anti-galectin-3 antibodies.

#### Fluorescene measurements

Fluorescence measurements were performed using a SpectraMax M5 (Molecular Devices) in 96-well plates with total reaction volume of 100 µl in constant temperature of 37°C. All measurements were performed in duplicate. Excitation was set on 480 nm, and emission was set on 530 nm (detection of FITC fluorescence) with low PMT sensitivity. FITC–annexin A5 (1:25 dilution from stock solution) was added to the LUV suspensions (500 µM) with different phospholipid composition, supplemented with CaCl_2_ (5 mM) and divided in 100 µl samples in a 96-well plate. Cinnamycin (10 µM) or DMSO was added to the plate and FITC fluorescence was recorded every minute using the kinetic measurement mode of the instrument. Next, proteinase K was added to the wells for a further 50 min incubation and FITC florescence was recorded. Finally, Triton X-100 (0.5%) was added to the proteolysis samples.

### Protein interaction as determined by microscale thermophoresis

The interaction between cinnamycin and annexin V was measured by microscale thermophoresis using the Nanotemper Monolith NT.115 instrument. A constant amount of labelled recombinant annexin A5 (20 µg/ml) conjugated to Alexa Fluor 647 was mixed with a different amount of cinnamycin (from 7×10^−8^ mM to 1 mM) for 1 h at room temperature. Samples were then loaded onto capillaries and analysed with the Monolith NT.115. As a positive control of interaction, a constant amount of labelled annexin A5 (20 µg/ml) conjugated to Alexa Fluor 647 was mixed with a different amount of unlabelled recombinant annexin A5 (from 7×10^−6^ µg/ml to 100 µg/ml) for 1 h at room temperature in the presence of 5 µM of Ca^2+^ (CaCl_2_).

### Cinnamycin treatment and EDTA assay in cells

Cells were treated from 30 min to 1 h at 37°C with 1 µm cinnamycin (diluted in serum-free medium, SFM) or DMSO, used as a control. Cells were then washed twice with SFM and incubated with versene (EDTA solution) or SFM, used as a control, for 10 min at 37°C. The supernatant was collected, floating cells pelleted at 300 ***g*** for 5 min before filtering through a 0.2 µm syringe filter. The a sample of clarified supernatant was then mixed with 4× sample buffer [50 mM Tris-HCl pH 6.8, 2% SDS (w/v), 0.1% Bromophenol Blue, 10% glycerol and 100 mM DTT] and boiled for 5 min. Cells were lysed in lysis buffer (20 mM Tris-HCl pH 6.8, 137 mM NaCl, 1 mM EDTA, 1% Triton X-100 and 10% glycerol) at 4°C for 10 min and insoluble material removed by centrifugation at 10,000 ***g*** for 10 min at 4°C. Sample buffer was added and cell lysates were boiled (as above). Cell lysates and cell supernatants were then subjected to SDS-PAGE.

### Western blotting

All samples were resolved by 12% SDS-PAGE and transferred to polyvinylidene difluoride membranes for blotting. Membranes were blocked with 0.05% (w/v) skimmed milk powder in PBS containing 0.1% Tween-20 (PBS-Tween) for 30 min at room temperature. Membranes were then probed with an appropriate dilution of primary antibody overnight at 4°C. Membranes were washed three times in PBS-Tween before incubation in diluted secondary antibody for 1 h at room temperature. Membranes were washed as above and developed via ECL (Amersham ECL Western Blotting Detection Reagent RPN2106 for the detection of proteins in the cell lysates or Cyanagen, Westar XLS100 for the detection of proteins in the eluate fractions) using a BioRad Chemi Doc XRS system. Membranes were stripped with Restore plus (Thermo Fisher Scientific, 46430) as per the manufacturer's instructions.

### Lentiviral transfection

HEK293FT packaging cells growing in 10-cm dishes were transfected with a mix of 11.68 μg packaging vector (psPAX2), 5.84 μg envelope vector (pMD2.G) and 18.25 μg ANO6-Plvx-mCherry-c1 vector. Polyethylenimine (PEI) was used as transfection reagent. At 48 h after transfection, cell culture medium was collected and replaced by fresh medium; this step was repeated two times at intervals of 24 h. Virus preparations were then combined. Viral particles were added to cells, which were spun at 1000 ***g*** for 30 min and incubated overnight. After 24 h, medium was replaced by new medium and cells were incubated for 5 more days. Transduced cells were selected with puromycin and sorted to enrich for mCherry-expressing cells.

### LDH assay

The lactate dehydrogenase (LDH) assay was performed according to manufacturer's instructions (Thermo Fisher Scientific, 88953).

### Mass spectrometry

Samples were submitted to the Cambridge Institute for Medical Research–Institute of Metabolic Science proteomics facility where they were analysed using a Thermo Orbitrap Q Exactive with an EASY-spray source and Dionex RSLC 3000 UPLC.

### TMEM16F CRISPR-mediated gene disruption

TMEM16F was targeted in either exon 2 or exon 3, both of which are conserved across splice variants. TMEM16F-specific oligonucleotides (Sigma-Aldrich; Table S1) were designed ,and top and bottom strands were annealed, and then cloned into the Cas9 expression vector pSpCas9(BB)-2A-Puro (PX459) (Addgene plasmid #48139) as previously described (Ran et al., 2013). Transfected cells were selected with 2.5 µg/ml puromycin for 24 h. Once recovered, cells were single-cell sorted into 96-well plates by fluorescence activated cell sorting (FACS). TMEM16F targeting was verified by collecting genomic DNA from clonal lines using the QuickExtract DNA extraction solution, and the CRISPR/Cas9 targeted region amplified with primers flanking at least 200 base pairs either side of the expected cut site (Table S1). PCR products were sequenced by Sanger sequencing and insertions and deletions analysed by using the Tracking of Indels by DEcomposition (TIDE) web tool (Brinkman et al., 2014). Additionally, to analyse insertions and deletions larger than 50 base pairs, code, kindly provided by Prof. Bas van Steensel (The Netherlands Cancer Institute), was used. TIDE analysis showed that the expected region had been targeted and each knockout clone was devoid of wild-type TMEM16F DNA (Fig. S2A,B). Clonal wild-type control lines that had been through transfection, selection and the single-cell cloning steps, but had not efficiently targeted TMEM16F, were used as matched positive controls for each exon (Fig. S2A,B). Oiwng to the lack of antibodies specific for TMEM16F, we were unable to analyse expression at the protein level; therefore, we assessed the mRNA levels, which were reduced in TMEM16F-knockout cells (Fig. S2C).

### Ionomycin and cinnamycin PS flow cytometry assay

Approximately 1×10^6^ HeLa cells in six-well plates were washed once in serum-free medium, incubated in versene (EDTA solution) at 37°C until they had detached and collected in to an excess volume of complete DMEM. Cells were pelleted at 300 ***g*** and resuspended in 500 µl annexin A5 binding buffer (Abcam). Cells were then transferred into FACS tubes containing 5 µl recombinant annexin-A5–Cy5 and 1 µl propidium iodide and ether 1 µl ionomycin (10 µM final concentration) or ethanol. Cells were carefully mixed and incubated at 37°C for 10 min only. Cells were immediately analysed on a FACSCalibur (BD) instrument equipped with lasers providing 488 nm and 633 nm excitation sources. Annexin-A5–Cy5 fluorescence was detected in the FL4 detector (661/16 BP) and propidium iodide was detected in the FL2 detector (585/42 BP). For analysis of cinnamycin, cells were collected into annexin A5 binding buffer as above and incubated with 1 µM cinnamycin or the equivalent volume of DMSO at 37°C for 50 min. Annexin-A5–Cy5 and propidium iodide were then added and cells incubated at 37°C for a further 10 min. Cells were analysed by flow cytometry immediately, as described above.

### Cell sorting

For sorting, cells were collected with trypsin/EDTA, washed and florescence activated cell sorting (FACS) was carried on an Influx cell sorter (BD) or Aria-Fusions (BD) equipped with lasers providing 488 nm and 640 nm excitation sources. mCherry fluorescence was detected in 610/20 BP detector on Influx and Aria Fusion instruments.

### Statistical analysis

Significance levels for comparisons between groups were determined with a two-sample Student's *t*-test.

## Supplementary Material

Supplementary information
