# Supplementary material for: Transbilayer phospholipid movement facilitates the translocation of annexin across membranes
Source: J Cell Sci. 2018 Jul 19;131(14):jcs217034. doi: 10.1242/jcs.217034 (PMC6080606; doi:10.1242/jcs.217034)
Supplement: Supplementary information [file joces-131-217034-s1.pdf]

Supplementary Figures:

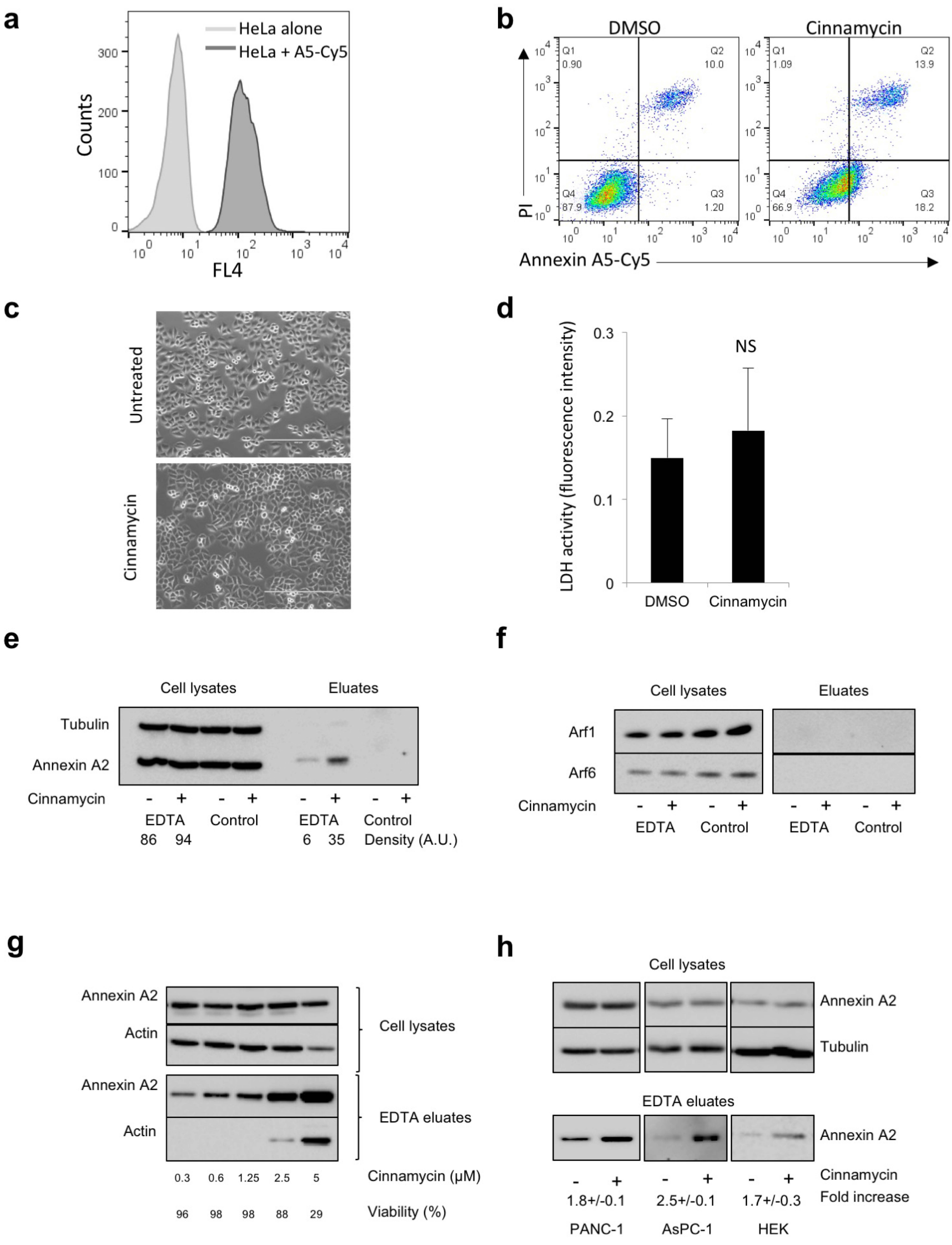

Journal of Cell Science • Supplementary information

Supplementary Figure 1: Cinnamycin increases cell surface annexin A2.

(a) Histogram of live cells incubated with recombinant annexin A5-Cy5 or untreated **(b)** Raw data of the flow cytometry analysis showing annexin A5-Cy5 and propidium iodide (PI) staining from the experiment described in Fig. 1a. **(c)** Phase contrast pictures of HeLa cells treated with cinnamycin for 30 min. **(d)** Lactate Dehydrogenase activity in the eluates of HeLa cells treated with cinnamycin for 30 min. Error bars represent ±s.e.m. from biological replicates (n = 2); NS: not significant. **(e)** Western blotting analysis of cell lysates and eluates of HeLa cells treated with cinnamycin (30 min at 37°C) and then with EDTA (10 min at 37°C) as indicated. The same volume of sample was loaded to assess direct comparison between lysates and eluates. **(f)** Western blotting analysis of cell lysates and eluates of HeLa cells treated with cinnamycin (30 min at 37°C) and then with EDTA (10 min at 37°C) as indicated. **(g)** Western blotting analysis of cell lysates and eluates of HeLa cells treated with different concentrations of cinnamycin (30 min at 37°C) and then with EDTA (10 min at 37°C) as indicated. Viability was measured by trypan blue assay. **(h)** Western blotting analysis of cell lysates and eluates of PANC-1, AsPC-1 and HEK-293 cells treated with cinnamycin (30 min at 37°C) and then with EDTA (10 min at 37°C) as indicated. Quantification of cell surface annexin A2 is shown (fold change measured as band intensity [cinnamycin(eluate/lysate)/DMSO(eluate/lysate)]). Error bars represent ±s.e.m. from biological replicates (n = 3); * $p<0.05$.

Journal of Cell Science • Supplementary information

a

Clone Genotype	Exon	TIDE efficiency	INDELs (TIDE)			
A5 - wild type	E2	5%	Zero 95%			
F6 - knockout	E2	94.2%	NEG 13 21.6%`	NEG 11 26.9%	NEG 7 21.7%	PLUS 1 23.9%
G9 – wild type	E3	4.3%	Zero 94.90%			
E7 - knockout	E3	73.6%	NEG 5 25.3%	NEG 2 26.6%	PLUS 1 13.1%	Possible large INDEL over 80 bp

b

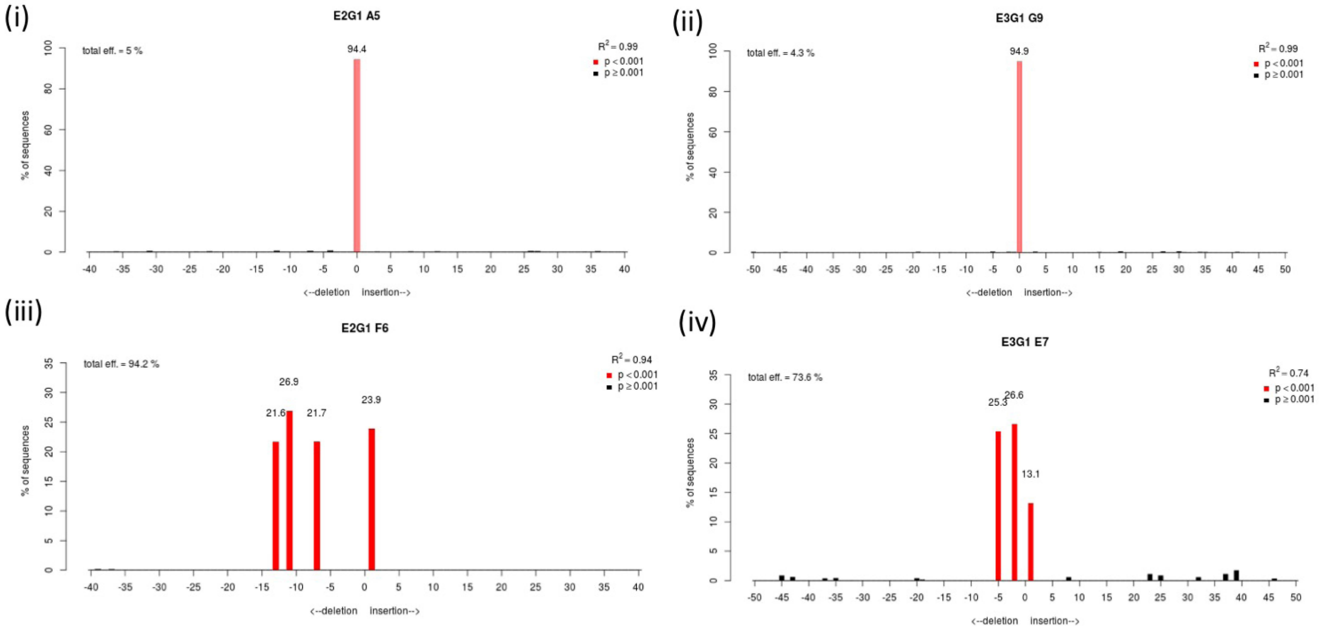

c

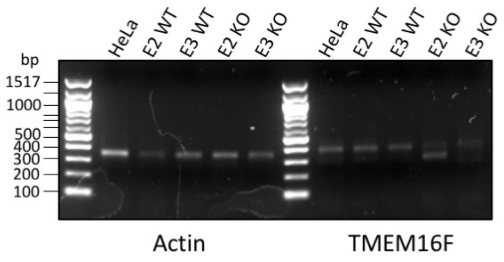

Supplementary Figure 2: Generation of TMEM16F knockout cells.

(a) Tracking of Indels by Decomposition (TIDE) analysis of TMEM16F CRISPR targeted clone summary. **(b)** Tracking of Indels by Decomposition (TIDE) analysis (i) sgRNA targeting TMEM16F exon 2, 94.4% sequence wild type; (ii) sgRNA targeting TMEM16F exon 3, 94.9% sequence wild type; (iii) sgRNA targeting TMEM16F exon 2, 0% sequence wild type; (iv) sgRNA targeting TMEM16F exon 3, 0 % sequence wild type. **(c)** Reverse transcription PCR for TMEM16F expression. RNA from TMEM16F clones analysed for TMEM16F mRNA.

Journal of Cell Science • Supplementary information

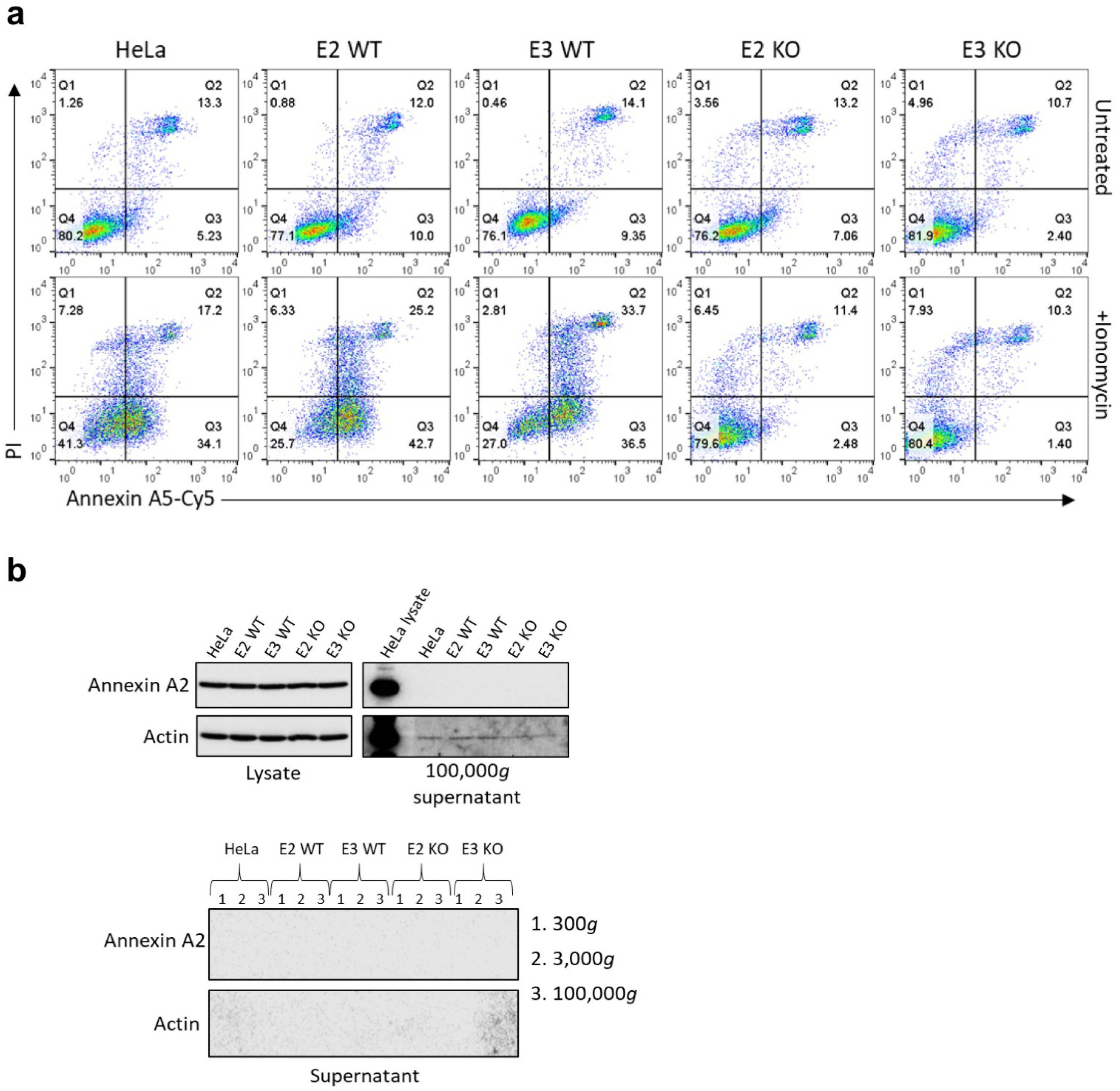

Supplementary Figure 3. TMEM16F knockout cells do not externalise PS or secrete Annexin A2.

(a) Raw data of the flow cytometry analysis showing recombinant annexin A5-Cy5 and propidium iodide (PI) staining from the experiment described in Fig. 4a. **(b)** Annexin A2 is not secreted from TMEM16F-deficient cells. Cells were seeded at 70% confluence and incubated in SFM for 24 h at 37°C. The medium was collected, cells and debris removed by centrifugation and analysed for annexin A2 by western blotting (*n*=3).

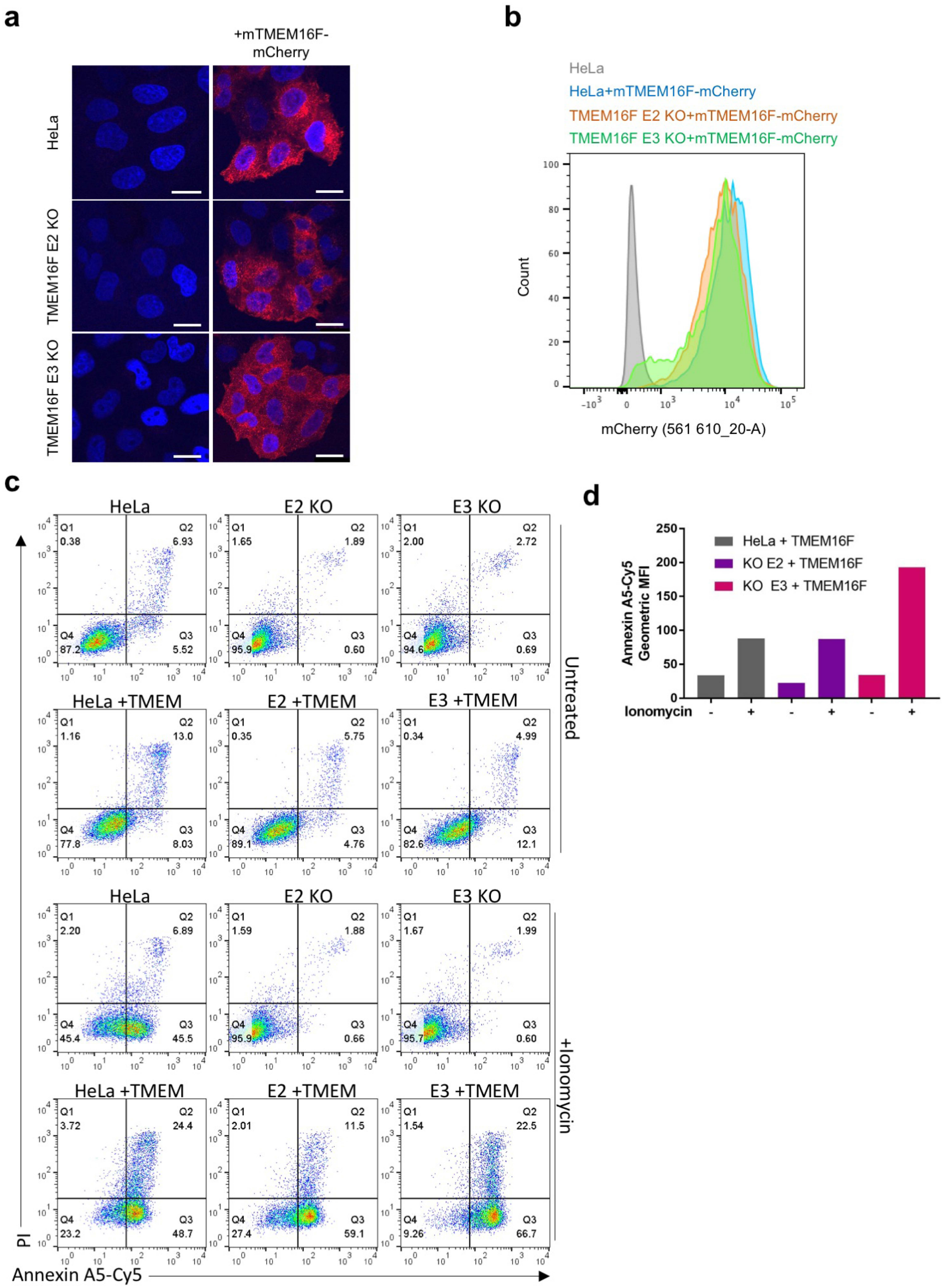

Journal of Cell Science • Supplementary information

Supplementary Figure 4. mCherry-TMEM16F expression rescues lipid movement.

(a) Subcellular localisation of mCherry-mTMEM16F. In both wild-type and TMEM16F-deficient cells mCherry-TMEM16F localises to the perinuclear location and to the plasma membrane as seen by confocal microscopy analysis of mCherry expression. **(b)** Flow cytometry analysis of mCherry-mTMEM16F expression in wild-type and TMEM16F-deficient cells. **(c)** Raw data of the flow cytometry analysis showing recombinant annexin A5-Cy5 and propidium iodide (PI) staining from the experiment described in Fig. 5b. **(d)** Expression of mCherry-mTMEM16F rescues lipid externalisation in TMEM16F-knockout cells. Wild-type and TMEM16F-knockout Hela cells expressing mCherry-mTMEM16F were treated with ionomycin and analysed for annexin A5-cy5 binding. Representative results shown as the expression level of mCherry-mTMEM16F varied in 3 separate experiments.

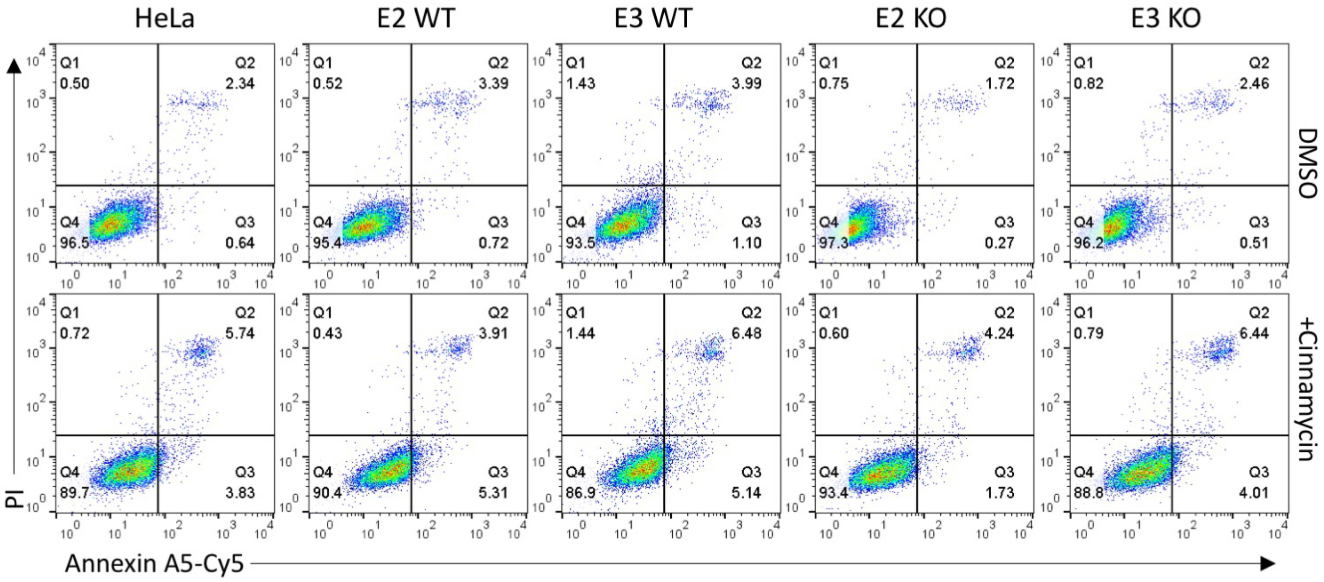

Supplementary Figure 5. Cinnamycin rescues lipid movement.

Raw data of the flow cytometry analysis showing recombinant annexin A5-Cy5 and propidium iodide (PI) staining from the experiment described in Fig. 6a.

Journal of Cell Science • Supplementary information

Supplementary Table 1. Oligonucleotides used for TMEM16F CRISPR/Cas9 targeting, amplification of targeted area and sequencing.

Primer sequence (5’-3’)	Target	Function
CACCGgtcagcatgattttcgaaccc	TMEM16F Exon 2	Primer pair for cloning into pX459 CRISPR<br>Note: Bold sequence for cloning
AAACgggttcgaaaatcatgctgacC		
CACCGgaacaaagtcaattcttcgc	TMEM16F Exon 3	Primer pair for cloning into pX459 CRISPR<br>Note: 3’-5’ sequence, bold sequence for cloning
AAACgcgaagaattgactttgttcC		
GGCATCGTCTGGTAGATCCAA	TMEM16F Exon 2	Forward sequencing primer
ACACAATCAAGTTCAGCAACCT	TMEM16F Exon 2	Reverse sequencing primer
TCCTTTCACCAAGAATAGACCATGT	TMEM16F Exon 3	Forward sequencing primer
AGAGCCTGAGGAAGTTTCTGAT	TMEM16F Exon 3	Reverse sequencing primer
GACATGAAAAAGATGAGCAGGAA	TMEM16F Exon 1	RT PCR Forward primer
CTCCCATGGTGCGTGTACTT	TMEM16F Exon 5	RT PCR Reverse primer

Journal of Cell Science • Supplementary information
